# Workplace age discrimination and engagement: The role of emotional regulation

**DOI:** 10.1192/j.eurpsy.2023.546

**Published:** 2023-07-19

**Authors:** I. Miguel, S. Silva, S. von Humboldt, P. Tavares, G. Low, I. Leal, J. Valentim

**Affiliations:** ^1^Portucalense Institute of Human Development & Department of Psychology and Education, Portucalense University, Porto; ^2^ISPA - Instituto Universitário, Lisbon, Portugal; ^3^William James Center for Research, ISPA - Instituto Universitário; ^4^Advance/CSG, ISEG Lisbon School of Economics and Management, Universidade de Lisboa, Lisbon, Portugal; ^5^Faculty of Nursing, University of Alberta, Edmonton, AB, Canada; ^6^Faculty of Psychology and Educational Sciences, University of Coimbra, Coimbra, Portugal

## Abstract

**Introduction:**

Gaining competitive advantage is essential to modern organizations, for which it is fundamental that workers are engaged with their work. Perceived age discrimination in the workplace is a factor that may influence workers’ engagement.

**Objectives:**

The present study aimed to analyze the moderating role of emotional regulation in the relationship between perceived age discrimination and work engagement.

**Methods:**

This empirical study included a sample of 452 Portuguese workers of various age groups, between 18 and 65 years-old and used the questionnaire as data collection method.

**Results:**

Results show that perceived workplace age discrimination negatively impacts work engagement. Further, results suggest that emotional regulation exacerbates the negative relationship between perceived age discrimination and work engagement.

**Conclusions:**

Age management strategies to address perceived age discrimination and work engagement, particularly due to the increasing proportion of older workers, are discussed.

**Disclosure of Interest:**

None Declared

